# Children and bioethics: clarifying consent and assent in medical and research settings

**DOI:** 10.1093/bmb/ldac038

**Published:** 2023-02-01

**Authors:** Merle Spriggs

**Affiliations:** Children’s Bioethics Centre, Melbourne School of Population and Global Health, University of Melbourne, Parkville, VIC 3052, Australia; Biomedical Ethics, Murdoch Childrens Research Institute, Parkville, VIC 3052, Australia

**Keywords:** bioethics, children, informed consent, assent, dissent, competence, capacity, decisional authority

## Abstract

**Introduction:**

The concept of consent in the pediatric setting is complex and confusing. Clinicians and researchers want to know whose consent they should obtain, when a child can provide independent consent and how that is determined. The aim of this article is to establish what produces the justification to proceed with medical or research interventions involving children and the role of consent in that. I clarify concepts such as consent, assent, capacity and competence.

**Source of data:**

Literature review.

**Areas of agreement:**

Engaging with children and involving them in decisions about matters that affect them is a good thing.

**Areas of controversy:**

The role of competence or capacity and the question of when a child can provide sole consent.

**Growing points:**

Flawed assumptions around competence/capacity.

**Areas for developing research:**

An account of children’s well-being that accommodates children’s interests during the transition to adulthood.

## Introduction

A child’s agreement or ‘consent’ to medical treatment or research participation does not have the same moral weight as the agreement or consent of an adult. The voluntary informed consent of an adult to undergo medical treatment or to participate in research is the justification to proceed. The difference when the patient or research participant is a child, is that children have limited cognitive abilities and maturity. Children need someone else to represent their interests. This is a temporary situation, however. Children develop over time and take on an increasing role in decisions about the things that affect them.^1^

**Fig. 1:**
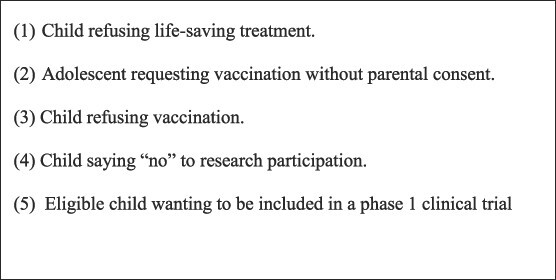
Scenarios where questions of consent arise.

The purpose of this paper is to describe and analyse consent in the pediatric setting. This involves questions about the function and value of ‘consent’ (of the parent and the child). I establish what it is that produces the justification to proceed with medical or research interventions involving children and the role of consent in that. My focus is situations where questions of consent arise, such as research participation, a child requesting vaccination without parental consent or a child refusing treatment or vaccination—see Figure 1. I identify flawed assumptions and clarify commonly confused concepts such as consent, assent, capacity, competence, autonomous decisions and decisional authority.

## What constitutes consent in the pediatric setting?

In the health setting, consent, specifically informed consent, is important. Based on the literatures of medicine, research ethics, law, philosophy and psychology, informed consent presupposes competence (in decision-making) and includes the ‘crucial elements’ of (i) disclosure, (ii) understanding and (iii) voluntariness.^2^ A definition of informed consent derived from these elements is as follows: ‘A person gives an informed consent to an intervention if (and perhaps only if) he or she is competent to act, receives a thorough disclosure, comprehends the disclosure, acts voluntarily and consents to the intervention’.^2^ For children, there is ‘no consensus’ as to how the elements of informed consent should be applied or modified.^3–6^ In adults, the ‘primary justification’ or underlying ethical value of informed consent is autonomy.^2^ For interventions involving children, the underlying ethical value is respect for persons, i.e. attending to the welfare or interests of the child (beneficence).^2^

In the pediatric setting, the concept of consent (of the parent and the child) can be confusing for clinicians and researchers. Key questions that arise are whose consent should they obtain to authorise an intervention and when can a child provide independent consent.

The focus of this paper is ethical and conceptual issues rather than the law. The law on what constitutes consent (or refusal) in the pediatric setting varies from place to place and continues to evolve.^6^ Clinicians and researchers need knowledge of relevant laws, but they also need a clear understanding of the terms and concepts used to draw conclusions or justify medical or research interventions involving children. A key purpose of this paper is to help clinicians and researchers be clear about the terms and concepts they use in their ethical deliberations.

### When is consent an issue?

In the research setting, consent for a child to participate is ‘mandated’.^6^ Parent/guardian ‘consent’ is required together with the child’s agreement or assent.^7^ In the clinical setting, consent is required prior to any substantial intervention, but questions about children’s consent (separate to that of parental consent) usually only arise in exceptional circumstances such as when a child seeks or refuses treatment independently of others.^8^

## Proxy consent

Parents are regarded as appropriate decision-makers for their children.^1^ It can be presumed that they will ‘do a better job’ than anyone else because in most cases, they ‘care deeply about’ and know their children and their children’s interests ‘better than others do’.^9^ Parental or proxy consent follows the same process and is held to the same standard of disclosure as voluntary informed consent^10^^,^^11^; however, parental or proxy consent is not the same as ‘real consent.’^12^ The underlying value is not autonomy or parental rights. It is about serving children’s welfare, not preserving parental autonomy.^1^^,^^12^ In situations where parental consent is waived, an alternative form of protection is needed.^10^^,^^11^ The functions of parental consent include (i) the exercise of ‘parental responsibility’^1^, (ii) protection for the child^3^^,^^10^ and (iii) ‘a check on the power’ of the person seeking consent.^12^ The term ‘parental permission’ is sometimes used rather than parental consent.

## Assent

Children who cannot consent for themselves or protect their own interests are still owed moral consideration. This is where the concept of assent comes in. A requirement for assent demonstrates respect for the child as a person. Rudimentary definitions of assent include: ‘agreement with proposed interventions’^1^; and for the research setting: ‘affirmative agreement to participate’ and ‘mere failure to object’ is not deemed assent.^13^ These stipulative definitions differ from dictionary definitions. Although not specifically stated, an obvious aspect of assent is children’s involvement (however limited) in discussions.

There is some opposition to the term ‘assent’. According to one bioethicist, we should abandon the term altogether and replace it with something like ‘developmentally appropriate involvement in decision making’ to better capture what we are hoping to achieve.^14^ This is a criticism of the term rather than the concept. Nevertheless, the term ‘assent’ is well established in the literature and in research ethics guidelines, so it makes sense to try to define what it is, what it is not, its value and its function.

A key feature of child assent is that it does not need to be burdened ‘with the same informational and decision-making standards as adult informed consent’.^10^ Assent is not to be relied on to protect the child. Parental consent/permission provides the protection. It is important, therefore, that assent should ‘only be understood in conjunction with’ parental consent/permission.^4^

Attempts to specify what is required for assent can end up conflating assent with adult informed consent,^4^ but the function as well as the underlying ethical value of assent is different. Respect for autonomy does not apply to children who lack autonomy. It is important to note, however, that the ethical value of respect for persons encompasses children *and* their developing autonomy.

Assent provides children with ‘the opportunity to choose to the extent that they are able’.^4^ It gives recognition to the value of the role for children ‘that lies between no involvement in discussions and full decisional authority.’^15^ This captures the idea that children, even very young children, benefit from knowing what will happen, having a say and being listened to, even though they do not have decisional authority.^15^

### Assent in the clinical setting

The literature on pediatric assent relating to research is more prolific and more developed than assent for the clinical setting. However, a recent article adds to the understanding of assent in clinical care by incorporating the moral value of children’s preferences. Navin and Wasserman describe a ‘preference’ in pediatrics as ‘an at least somewhat stable communicated desire for one intervention or outcome over another’. The key idea is that children’s preferences about medical treatment ‘always have some moral weight’—even though not always authoritative.^16^ There are situations in which ‘treating children against their preferences’ is the ‘right thing to do’ but, according to Navin and Wasserman, that ‘always involves an uncompensated moral loss’ which needs to be acknowledged with an apology or expression of regret.^16^ This account of assent fits well particularly for young children, and it accommodates children with intellectual disability.

### Assent as an opportunity for children to develop autonomy

The assent process is sometimes attributed value in the sense that involvement in decision-making helps ‘promote children’s moral growth and developing autonomy’.^4^ It allows, amongst other things, an opportunity ‘to obtain needed practice in making decisions’, contributes to children’s perception that ‘they have some control over their lives, and results in a greater sense of self-esteem and competence, while reducing anxiety’^11^ These positive side-effects or by-products of respecting the child as a person are appropriate and relevant, particularly in pediatric clinical decision-making. The research setting however is not the same. Viewing children’s involvement in discussions about experimental procedures that are not necessary for their care as an *opportunity* for them to benefit, seems misplaced. There are less fraught contexts in which children and their parents can seek out opportunities to promote decision-making skills and moral development.^17^

### Dissent: an aspect of assent

The requirement for assent in the research setting provides an opportunity for the adults in charge to take account of a child’s objections or distress—sometimes referred to as ‘dissent’.^1^^,^^11^ A child’s dissent carries substantial weight and requires a lesser level of understanding, if any.^10^ Even children who are ‘too immature’ to provide assent, may be able to register ‘an expression of disapproval.’^7^ For these children, waiving assent or overriding dissent is justified only in ‘exceptional circumstances’ such as where an ‘intervention or procedure’ offers ‘direct benefit that is important to the health or well-being of children and is available only in the context of the research.’^1^^,^^7^^,^^11^ For older children, refusal, like that of any other research participant, should be respected.^15^

Dissent is an aspect of assent. In pediatric clinical practice, assent is a ‘much more subtle and complex concept’,^3^—it demonstrates respect ‘even if that extends only’ to allowing children ‘to voice their dissent’.^3^ Disregarding a child’s dissent is arguably easier to defend in the clinical setting because pediatrics ‘systematically involves’ young children refusing or objecting to interventions that clinicians are ‘ethically justified or even ethically obligated to impose’.^16^ Nevertheless, justification for disregarding or coercively imposing upon a child’s objections generates different concerns at different stages of children’s development. The ‘deliberate objection’ of an adolescent ‘is to be distinguished from the behavior of an infant likely to cry or withdraw in response to almost any adverse stimulus’^7^ Both deserve moral consideration, but the objection of the adolescent is likely to carry more weight.

In both clinical and research settings, a psychological or developmental approach to child assent (and dissent), such as that proposed by Miller and Nelson, could help us understand how children can be meaningfully involved in decision-making,^4^ and the differing weights we attribute to children’s preferences across development. Miller and Nelson suggest consideration be given to ‘cognitive and non-cognitive variables’ that influence or contribute to children’s involvement in discussions—things such as ‘biological maturation, level of experience, and types of experiences.’^4^ They also include the idea that shared decision-making with parents and children taking on a greater role in decision-making over time, is ‘a characteristic of normal development.’^4^ This sort of approach is useful in helping us to understand how children can ‘choose to the extent that they are able’^4^ and to recognise why some expressions of dissent carry more weight. It steers us away from the trap of judging children’s decision-making capacities against adult standards.

**Fig. 2:**
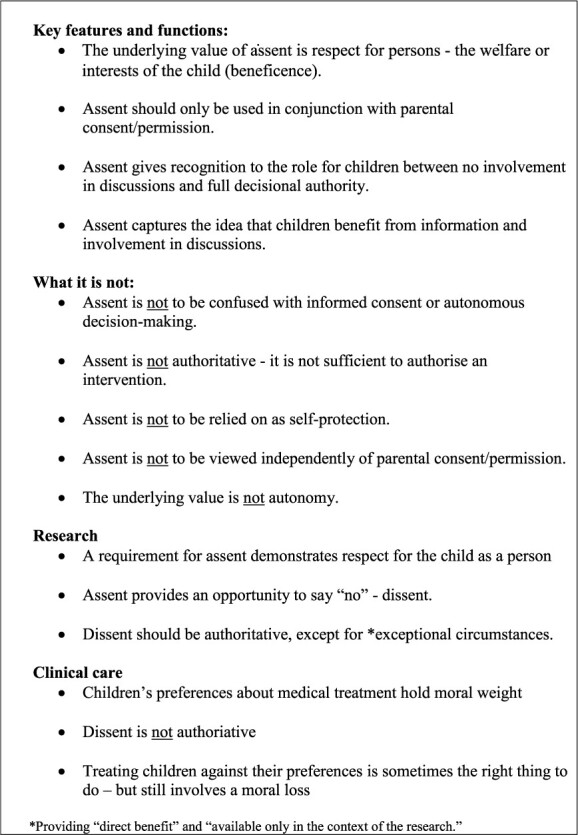
Assent (and dissent).

### In summary

It is useful to set out the parameters of assent (and dissent), with a list of key features and ethical functions—see Figure 2. This can assist in distinguishing what is at stake and help identify what it is that produces the justification to proceed with medical and research interventions involving children.^15^ We see also that although some of the key features and functions are identical for the clinical and the research settings, there are also differences.

## The significance of differences in research and clinical settings

Differences in the research setting and the clinical setting can alter the weight given to a child’s agreement or refusal. Research and treatment have different objectives, justifications and procedures. In the clinical setting, clinicians and patients share the same objective; the goal of a treatment, procedure or therapy is for the benefit of the patient. Research, on the other hand, is about the pursuit of knowledge with the aim of benefitting future people. Research may involve procedures (tests, needles, questionnaires) done primarily as part of the research. Even though researchers have ethical responsibilities to participants, they also have a competing interest in sticking to the protocol and completing the research.^18^ It can be difficult for research participants (and parents) to understand that research participants are treated differently than if they were simply a patient. This ‘misconception’ is widely recognised. Potential participants ‘inaccurately’ attribute ‘therapeutic intent to research procedures,’ believing that they will be offered the ‘same individually focused treatment’ they would receive in the clinical setting.^19^

Decisions about research can be more complex. Risks can be greater because research procedures are experimental—outcomes are unknown or untested. Also, the likelihood or seriousness of some risks are not easily determined.^3^ Although disclosure of benefits, risks, side-effects and alternatives are of equal importance in the clinical/treatment setting, it is reasonable to argue that consent in the clinical setting is a more ‘benign process’ with patients and clinicians sharing the same therapeutic objective.^18^ Risks are not ‘gratuitously undertaken’ as they are in research.^20^

Where children are concerned, there is additional complexity. Children have an unequal relationship with adults in which they are ‘generally less able’ to question, challenge or resist what adults propose^21^—and researchers have a potential conflict of interest in recruiting participants into their research. Children can be influenced or pressured by the real or perceived expectations of adults.^22^

What this all means, is that when consenting to or authorising research participation, potential participants, or parents, often require additional information and a greater level of understanding. They need an awareness of the ways in which research participation differs from clinical care—specifically in terms of risks, competing interests that come with the role of the researcher, and the potential loss of individually tailored care. They need to understand that research interventions are ‘neither necessary for their care nor perhaps fully understood’ and that what they are consenting to is participation ‘in the furthering of knowledge.’^20^

## Agreement and controversy

Despite difficulties in trying to apply informed consent or something of equivalence, in the pediatric setting, an area of agreement is that engaging with children and involving them in decisions about matters that affect them is seen as a good thing. This corresponds with the ideas captured within the concept of assent.^1^^,^^3^^,^^4^^,^^11^^,^^16^^,^^23^^,^^24^

The main area of controversy is the question of when children can provide sole consent. A widely held view is that children reach a point in their development where they can make their own decisions without the need for parental consent. These children are often referred to as ‘mature minors’^1^ or Gillick competent.^8^ Much of the disagreement around the question of when a young person should be able to make their own decisions centers around the capacities needed for voluntary informed consent. Some people argue that children can have the required capacities, while others argue that they do not.^24^^,^^25^A different view altogether is that in looking to questions about competence and capacities, we are asking the wrong question.^9^^,^^24^ I analyse the role of competence in children’s decisions in treatment and research settings, but first deal with an important issue around terminology.

## Competence or capacity to consent

### Terminology

Competence and capacity are often used interchangeably but some people prefer ‘capacity’ because they see it as a clinical determination and competence as a legal determination.^6^ Others argue that these distinctions break down and should not be relied on.^2^ In addition, there is a problem in the common practice of using the terms as a kind of shorthand. Competence has a ‘single core meaning,’ which is ‘the ability to perform a task’,^2^ and in the context of decision-making, it is clear what we mean when we say someone is competent or lacks competence. The term ‘capacity’ is different. People use the term ‘capacity’ in more ways, and as shorthand for capacity to consent, capacity to understand, capacity to be involved in decisions, cognitive capacity. Therefore, saying that a child has or lacks ‘capacity’ can have different meanings. If we use this term, it is important to specify how we are using it.

### The role of competence/capacity in pediatrics

A lot of attention is paid to children’s competence or capacity to provide consent—and even assent. Because we presume that adults are competent to decide about their medical treatment or research participation, the burden of proof is on anyone who suggests otherwise. Children, however, are presumed not competent to decide and that seems to raise a question about ‘how good their decision-making abilities’ must be to rebut that presumption.^9^ This leads to debates about whether adolescents have or do not have a level of understanding and cognitive capacity comparable to adults, with the assumption that if they do, they should be able to make their own decisions.^24^^,^^25^ We see that this reasoning entails a number of flawed assumptions: that a competent decision is necessarily an autonomous decision deserving of respect; that a competent or autonomous decision is always a good or wise decision; and that an autonomous decision is an independent decision made in isolation from others.^12^^,^^26^

There exists a body of criticism about the role and the prominence given to competence judgments in debates about children’s decision-making.^9^^,^^24^^,^^27–29^ The main criticism is that decision-making competence is conflated with the decision-making authority.^9^^,^^24^ Critics argue that a child’s decision-making competence is a necessary but not sufficient basis for giving decisional authority or for respecting the decisions they make.^9^^,^^24^^,^^29^ The relevant question seems not to be about whether adolescents ‘possess decision-making capacity’ but whether they should ‘be given decision-making authority’.^24^ Additionally, studies show that children want input from their parents and sometimes ‘prefer joint decision-making’.^4^ Putting this all together, one commentator succinctly states: ‘just because we *can* make competent decisions on our own doesn’t mean we *should*, or even that we actually *do*.’^24^ The mistake, according to Salter, is that we are looking for scientific answers to an ethical question when we look to scientific evidence about adolescents’ decision-making capacities.^24^

Likewise, Beauchamp and Childress claim that competence judgments are sometimes ‘incorrectly’ presented as ‘empirical’ judgments.^2^ Competence is not a value free concept. We may devise tests or standards (e.g. mature minor or Gillick competence tests) to assess competence, but value judgments determine how we use the test, how it is constructed and how it is used to sort persons into competent and incompetent.^2^

In distinguishing competence from decisional authority, prominent bioethicists Buchanan and Brock claim there is more than just the question of how good a child’s decision-making abilities must be to allow them to decide for themselves. Buchanan and Brock seek a ‘nonarbitrary’ way to determine that,^9^ and conclude that the answer is to balance ‘the major values at stake’^9^ which are (i) the child’s well-being, (ii) the child’s self-determination (i.e. ‘their interest in developing capacities to be self-determining adults’)^9^ and (iii) the parents’ interest in making decisions concerning their children.^9^ Buchanan and Brock go further still, claiming that a child’s competence is ‘even less decisive’ in the face of ‘legitimate third-party interests, such as the state’s interest in a healthy citizenry’.^9^

Finally, some people are troubled by the lack of a ‘bright line demarcating when a minor becomes “mature” enough to independently satisfy the decision-making criteria for informed consent or refusal.’^1^ It is seen as a problem that often requires legal input. According to Iltis, however, there is nothing ‘magic’ that happens when, for instance, someone turns 18—but ‘at some point’ we recognise people as having authority to make decisions for themselves ‘even if they are bad choices.’^25^ Salter argues that the bright-line threshold (e.g. for an age of majority) is only a problem if we think decision-making capacity is the deciding factor. In adults, we accept suboptimal decisions because adults ‘are fully responsible for themselves and the consequences of their medical decisions.’^24^

The problems with capacity and competence do not mean that there is no role for a child’s competence or capacity to understand or take part in decisions. The relevance is more likely as a guide to what information is appropriate to involve them in a meaningful way in decisions—‘to the extent that they are able’.^4^

## Scenarios

The purpose of the following scenarios is to canvas areas of agreement and controversy in the literature and determine what produces the justification to proceed and the role of consent and assent in that:

(i) Should a child’s refusal of life-saving treatment be overridden?

There is consensus in the literature that children ‘should not be allowed to refuse life-saving treatment, even when parents agree.’^1^ This differs from situations where a child has only a short time to live and the only treatment options are ‘unproven, overly burdensome and likely ineffective.’^1^ The key justification for overriding a refusal is the prevention of serious and avoidable harm, as well as preserving the child’s long-term interests (staying alive, becoming an autonomous adult) over their short-term interests.^6^^,^^8^

(ii) Can an adolescent consent to vaccination without parental consent/permission?

The justification to vaccinate an adolescent without parental consent is not based on the individual’s decision-making competence or in meeting the requirements of informed consent, even though the decision should be informed and voluntary. The issue is whether to grant decisional authority to the adolescent. The justification for granting decisional authority in this case is that it ‘facilitates access to a medically recommended and evidence-based treatment’; it involves ‘minimal personal risk, and offers substantial prosocial benefits’ including ‘community protection against the spread of dangerous and costly yet preventable diseases’.^30^ Granting decisional authority, circumvents harm from parental decisions based on misinformation or disinformation.^30^

(iii) Should we respect a child’s refusal to be vaccinated?

Refusal of vaccination by a child introduces the possibility of restraint—considered ‘an option of last resort’ but nevertheless ethically justified when vaccination is in the child’s interests.^31^ Age or stage of development plays a role. For a very young child, restraint (euphemistically referred to as ‘comfort holding’) may be ethically justified.^31^ For the same reason, sedation may be ethically justified for a child with developmental problems.^31^ For an older adolescent, however, the advice from the literature is that ‘persistent refusal should usually be respected.’^31^ Consideration is given to the young person’s ‘maturity and capacity to understand the nature’ of the decision they are making,^31^ but the decision to respect the refusal is not necessarily a reflection of the adolescent’s decision-making abilities. The prospect of physically restraining an adolescent (an almost adult) against their will surely looms large. In certain circumstances (e.g. a deadly pandemic), this scenario is similar to scenario 1.

(iv) Is a waiver of assent ethically justified in a phase 1 clinical trial?

Phase 1 clinical trials are necessary and important to find new treatments for children. The purpose of a phase 1 trial is to establish toxicity and the maximum dose that humans (specifically a child in this case) can tolerate. Children who are eligible and given access to these trials are terminally ill children who have exhausted all treatment options. The purpose is not to treat or cure. Any therapeutic benefit from a phase 1 trial is ‘incidental’ or ‘coincidental.’^32^ There is, therefore, no justification to override a child’s refusal to take part in a phase 1 trial.^17^ The weight of the child’s refusal is unrivaled. Additionally, forcing an unwilling child to take part in research becomes a professional issue—it would not be wise professionally, to enroll a child against their will in research that is not necessary for their care.

It is understandable that parents may still want their child to participate. When faced with the death of their child, parents’ fears and unrealistic hopes can dominate.^32^ Parents may ask that their child not be informed that they are in a trial. However, this does not justify a waiver of assent. Phase 1 clinical trials do not fit the description of an ‘exceptional’ circumstance where an intervention offers ‘direct benefit’ that is available only in the context of the research.”^7^^,^^11^ The issue here is not that hope cannot be a legitimate basis for parental consent, but that mistakes in thinking are not a legitimate basis, i.e. thinking that the trial is a form of medical treatment.^2^^,^^17^

(v) Should we respect a child’s request to be included in a phase 1 clinical trial

We may justify respecting an eligible child’s request to take part in a phase 1 clinical trial as an act of altruism, providing they understand two crucial things—(i) that any potential benefits will be for future children, and (ii) that they can withdraw from the trial at any time. Importantly, we need not assess the child’s competence to make this decision. If we grant the wishes of dying children in other things, and if the child is not being pressured or exploited, it seems reasonable to allow them the opportunity to help other sick children.^17^

## Conclusion

Parental consent and child assent are extremely important but there are circumstances in which they are not sufficient and other protections are needed—children’s welfare takes precedence. It is instructive that the scenarios discussed in this paper pose complex ethical dilemmas primarily because they involve children, not adults—and things go awry when we compare a child’s decision-making with that of a competent autonomous adult. This is not to dismiss the ethical value of respect for a child’s developing autonomy or considerations of a child’s capacity to understand or take part in decisions. These are captured in the ethical value of respect for persons—where there is an important role for child assent.

## Areas for developing research

The bioethics and philosophical literature of what constitutes a child’s well-being as opposed to an adult’s well-being is ‘in its infancy.’^33^ We need an account of children’s well-being that accommodates the transition to adulthood and reconciles a child’s current interests with their long-term interests.^34^

## Conflict of interest statement

None declared.

## Data availability

No new data were generated or analysed in support of this review.
